# Conditional Deletion of LRP1 Leads to Progressive Loss of Recombined NG2-Expressing Oligodendrocyte Precursor Cells in a Novel Mouse Model

**DOI:** 10.3390/cells8121550

**Published:** 2019-11-30

**Authors:** Ina Schäfer, Johannes Kaisler, Anja Scheller, Frank Kirchhoff, Aiden Haghikia, Andreas Faissner

**Affiliations:** 1 Department of Cell Morphology and Molecular Neurobiology, Ruhr University Bochum, Universitätsstr. 150, 44801 Bochum, Germany; Ina.Schaefer@rub.de; 2 Research Center Neuroimmunology, Department of Neurology, Ruhr-University Bochum, Universitätsstr. 150, 44801 Bochum, Germany; Johannes.Kaisler@ruhr-uni-bochum.de (J.K.); Aiden.Haghikia@ruhr-uni-bochum.de (A.H.); 3 Department of Molecular Physiology, Center for Integrative Physiology and Molecular Medicine (CIPMM), University of Saarland, Building 48, 66421 Homburg, Germany; Anja.Scheller@uks.eu (A.S.); Frank.Kirchhoff@uks.eu (F.K.)

**Keywords:** cre-recombinase, demyelination, experimental autoimmune encephalomyelitis (EAE), glial progenitor cells, myelin, tamoxifen

## Abstract

The low-density lipoprotein receptor-related protein 1 (LRP1) is a transmembrane receptor, mediating endocytosis and activating intracellular signaling cascades. LRP1 is highly expressed in the central nervous system (CNS), especially in oligodendrocyte precursor cells (OPCs). Previous studies have suggested LRP1 as a regulator in early oligodendrocyte development, repair of chemically induced white matter lesions, and cholesterol homeostasis. To circumvent embryonic lethality observed in the case of global LRP1 deletion, we generated a new inducible conditional knockout (KO) mouse model, which enabled an NG2-restricted LRP1 deficiency (NG2-CreERT2^ct2/wt^xR26eGFP^flox/flox^xLRP1^flox/flox^). When characterizing our triple transgenic mouse model, we noticed a substantial and progressive loss of recombined LRP1-deficient cells in the oligodendrocyte lineage. On the other hand, we found comparable distributions and fractions of oligodendroglia within the Corpus callosum of the KO and control animals, indicating a compensation of these deficits. An initial study on experimental autoimmune encephalomyelitis (EAE) was performed in triple transgenic and control mice and the cell biology of oligodendrocytes obtained from the animals was studied in an in vitro myelination assay. Differences could be observed in these assays, which, however, did not achieve statistical significance, presumably because the majority of recombined LRP1-deficient cells has been replaced by non-recombined cells. Thus, the analysis of the role of LRP1 in EAE will require the induction of acute recombination in the context of the disease process. As LRP1 is necessary for the survival of OPCs in vivo, we assume that it will play an important role in myelin repair.

## 1. Introduction

The low-density lipoprotein receptor-related protein 1 (LRP1) is a type-I transmembrane receptor. It consists of two covalently bound subunits, an 85 kDa intracellular α-chain and 515 kDa extracellular β-chain [1]. As a multifunctional receptor, LRP1 can bind a variety of up to 40 different ligands such as apolipoproteins (Apo), extracellular matrix molecules, and growth factors and is involved in their endocytosis [1,2]. Apolipoproteins, especially ApoE, mediate cholesterol transport in various cell types [3]. Beyond its endocytotic function, LRP1 is involved in intracellular signaling and can activate, for example, the ERK and AKT pathways [4,5,6].

The LRP1 receptor is widely expressed in the body (liver, lung, blood vessels) and particularly in the CNS [1,7,8]. Radial glia, neuroblasts, astrocytes, neurons, and especially oligodendrocyte precursor cells (OPCs) serve as main sources for LRP1 [9,10,11,12]. In the past, many studies have focused on LRP1 and demonstrated that a global deletion of the receptor leads to embryonic lethality [13]. Therefore, conditional *Lrp1*-knockout (KO) models have been developed. Previous studies have concentrated on LRP1 function in oligodendrocytes, the myelinating macroglia in the CNS. Originating from highly migratory and proliferative OPCs, which populate the forebrain, oligodendrocytes differentiate into mature cells in their target compartment. Due to cytoskeletal rearrangements, myelin membrane expansion of mature oligodendrocytes enables myelination, and thereby the electrical isolation of nerve fibers [14,15,16]. 

It has been shown that neural stem cells, which lack LRP1, have a significantly reduced potential to differentiate into OPCs, immature or mature oligodendrocytes, indicating a crucial role of LRP1 in oligodendrogenesis [10,11]. Accordingly, LRP1 has been found highly expressed in OPCs, whereas it is downregulated in mature, myelinating oligodendrocytes on both the mRNA and protein level [9,12,17]. This suggests a link between LRP1 and early oligodendrocyte development. Moreover, oligodendrocyte functions are affected by LRP1. Thus, LRP1 deletion in chemically induced white matter lesions revealed attenuated remyelination and compromised repair of white matter. Furthermore, cholesterol homeostasis is regulated by LRP1, which plays a key role in oligodendrocyte differentiation [16]. 

According to the preliminary data, we generated a new inducible, conditional KO mouse model with OPC-restricted LRP1-deficiency: NG2-CreERT2^ct2/wt^xR26eGFP^flox/flox^xLRP1^flox/flox^. NG2, together with PDGFRα, serves as a marker for OPCs [18,19], which enabled us to delete LRP1 in early oligodendrocyte development and analyze the ensuing effects of LRP1-deficiency on the whole oligodendrocyte lineage. The characterization of our novel model revealed similar distributions and fractions of oligodendroglia in the Corpus callosum. However, substantial and progressive loss of recombined LRP1-deficient oligodendrocytes was observed over time. Moreover, when experimental autoimmune encephalomyelitis (EAE) was elicited by immunization with a MOG peptide, the LRP1 KO animals were more strongly affected, with greater functional deficits in comparison to the control. On the cellular level, the in vitro myelination assay revealed elongated internodes in the LRP1 KO condition. Considering our findings, we propose that LRP1 is a critical long-term regulator of oligodendrocyte survival in vivo and suggest a crucial role of LRP1 for myelin quality. 

## 2. Materials and Methods

### 2.1. Ethics Statement

This study was carried out at the Ruhr-University Bochum with conformity to the recommendations of local guidelines in experimental animal handling. The animal experiments were approved by the “Landesamt für Natur, Umwelt, und Verbraucherschutz” (LANUV) in Recklinghausen, North Rhine-Westphalia with the reference number 84-02.04.2015.A149. The study referred to the 3Rs (Replacement, Reduction, Refinement) and tried to reduce animal numbers and refine experimental conditions. 

### 2.2. Generation of the New Mouse Model and Housing

The new mouse model was generated by cross-breeding previously described mouse lines. LRP1^flox/flox^ mice (B6;129S7-*Lrp1^tm2Her^*/J), which were obtained from the Jackson Laboratory, and the transgenic constructs have been previously published by Rohlmann et al. [20]. In NG2-CreERT2_NGCE_ mice, the inducible Cre DNA recombinase CreERT2 was knocked into the NG2 locus. Only heterozygous mice were used [21]. By crossbreeding the mice to homozygous floxed reporter mice [22], recombined cells could be identified by the genetically encoded GCaMP3 reporter expression that can be detected by anti-GFP antibodies. Since we did not take advantage of the Ca^2+^ indicating property of GCaMP3, we termed this reporter mouse line R26eGFP^flox/flox^. By crossbreeding the two different mouse lines, NG2-CreERT2^ct2/wt^xR26eGFP^flox/flox^xLRP1^flox/flox^ (KO) and NG2-CreERT2^wt/wt^xR26eGFP^flox/flox^xLRP1^flox/flox^ (control) animals were generated in one litter. Both received tamoxifen via the lactating mother.

For in vivo analysis, postnatal day (P)7, P14, P21, P28, P42, P56, and P56–70 littermates were analyzed. An in vitro myelination assay (Figure S1) required P6 to P9 animals. 

Animals were housed in an open cage system with 12 h-day/night-cycles and 25 °C room temperature. Water and diet were accessible ad libitum.

### 2.3. Genotyping

To genotype the littermates, genomic DNA from tail biopsies was used. All primers (Sigma, St. Louis, MO, USA), amplified product sizes, and sources are listed in Table 1 (base pairs: bp, for: forward, rev: reverse, WT: wildtype). 

### 2.4. Injection of Tamoxifen to Animals

**Figure 1:**
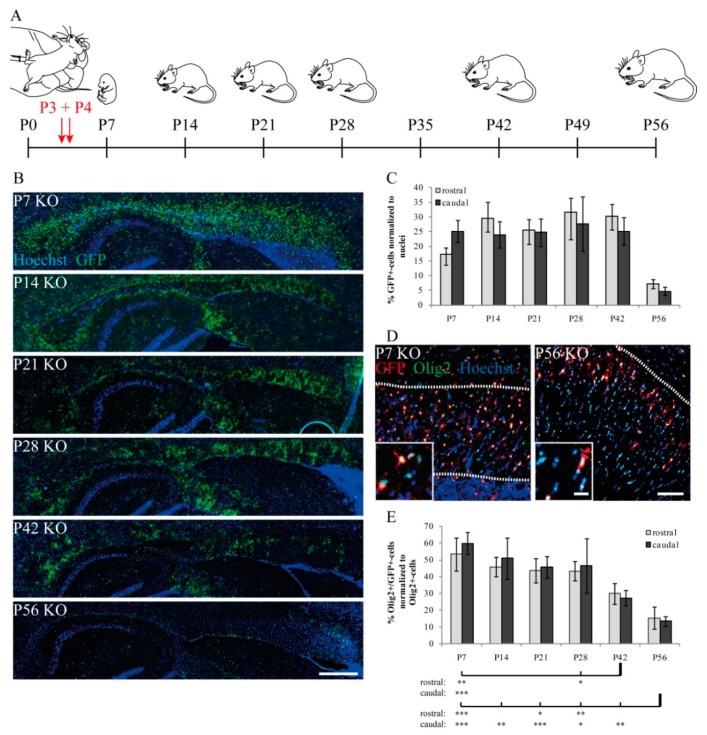
Recombination efficiency and portion of recombined cells over time. (**A**) Scheme of tamoxifen administration and analyzed ages of experimental animals. (**B**) Immunohistochemical stains against green fluorescent protein (GFP) indicating recombined cells within postnatal day (P) P7-P42 KO tissue (scale bars: 100 µm). (**C**) Quantification of GFP-expressing recombined cell fractions in the Corpus callosum of various analyzed stages. (**D**) Representative immunohistochemical staining against GFP and Olig2 (scale bars: 100 µm, 20 µm). (**E**) Quantification of double-positive cells for GFP and Olig2, defined as recombination efficiency, indicated an initial efficiency of more than 50%, before a significant and progressive loss of double-positive recombined LRP1 KO oligodendrocytes was observed over time. At P56, the loss of double-positive cells compared to earlier time points reached statistical significance, whereas this effect was not that prominent at P42 (*p* ≤ 0.05 for *, *p* ≤ 0.01 for **, and *p* < 0.001 for ***). Data are expressed as the mean ± SEM. N = 3–4, n = 9–12 per rostral and caudal part. At least 200–1200 cells per section were counted. Depending on normally or not normally distributed data, the Student’s *t*-test or Mann–Whitney U test was used for evaluation within the individual ages.

The KO was induced via administration of tamoxifen. Therefore, feeding mothers were intraperitoneally injected with 100 mg/kg tamoxifen (Sigma) dissolved in corn oil (Sigma), on two consecutive days (Figure 1A). Maternal tamoxifen metabolites reached the pups via milk at postnatal days (p)3 and 4 (referring to [21]). Animals were monitored and weighed every 2–3 days and evaluated with a clinical score. 

### 2.5. Decapitation, Perfusion, and Dissection

For the experiments, P7 animals were decapitated, whereas P14, P21, P28, P42, P56, and P56–70 animals were anesthetized (100 mg/kg ketamine (CP-Pharma, Burgdorf, Germany), 10 mg/kg Xylazin (CP-Pharma)), and perfused with PBS/heparin (Ratiopharm, Ulm, Germany). Therefore, a constant pressure of 0.7 mL/min was generated with a peristaltic pump. After perfusion for 10–15 min, the animals were dissected. For all ages, the brains were removed from the skull and separated into hemispheres for cryosections, PCR, and western blot analysis.

### 2.6. Cryosections

For cryosections, the dissected tissue was postfixed in 4% paraformaldehyde (PFA; Carl Roth GmbH and Co. KG, Karlsruhe, Germany) overnight and afterward drained with 20% sucrose (Fisher Chemical by Thermo Fisher Scientific, Bedford, MA, USA), again overnight. The tissue was embedded in tissue freezing medium (Leica Biosystems, Mount Waverly, Australia) to prepare sagittal cryosections (14 µm). Cryosections were stored at −20 °C until further use. 

### 2.7. Immunohistochemistry (IHC)

Intranuclear immunohistochemical staining started with incubation for 1 h in citrate buffer (solution A: 0.1 M citric acid-1-hydrate, solution B: 0.1 M Na-citrate-dihydrate; 1 mM of solution A and 4 mM of solution B in Aqua dest) at 70 °C. After three washes with PBS (10 × PBS: 137 mM NaCl, 3 mM KCl, 6.5 mM Na_2_HPO_4_•2H_2_O, 1.5 mM KH_2_PO_4_ in Aqua dest; 1 × PBS: 10 × PBS in Aqua dest, pH 7.4), blocking buffer (PBS, 0.1% Triton X-100, 1% bovine serum albumin (BSA, Sigma), 3% serum (Dianova GmbH, Hamburg, Germany)) was added to the slices for 1 h. The primary antibody was dissolved in blocking buffer and incubated on the sections overnight at 4 °C. Next, three washing steps with PBS followed and secondary antibody, dissolved in blocking buffer, was added for 2 h at room temperature. Finally, three washing steps in PBS and mounting with ImmuMount (Thermo Fisher Scientific) and coverslips were performed. Extranuclear immunohistochemical staining started with three PBS washes and followed the same protocol as described above. Antibodies: APC (clone CC1, 1:100, ab16794, Lot: GR322482-3, Abcam; Cambridge, UK), GFP (1:200, AB3080; Lot 2929345, Millipore by Merck, Darmstadt, Germany; 1:500, 600-101-215, Lot: 33301, Rockland Immunochemicals Inc., Limerick, PA, USA), LRP1 (1:500, ab92544, Lot: 6R259330-27, Abcam), Olig2 (1:400, AB9610, Lot: 3071572, Millipore), PDGFRα (1:300, sc-338, Lot: E2015, Santa Cruz Biotechnologies Inc., Dallas, TX, USA), Donkey α rabbit AF488 (1:250, 711-545-152, Lot: 127498, Jackson ImmunoResearch Laboratories Inc., West Grove, PA, USA), Donkey α rabbit Cy3 (1:500, 711-165-152, Lot: 130990, Jackson ImmunoResearch Laboratories Inc.), Donkey α mouse Cy5 (1:150, 715-175-150, Lot: 129945, Jackson ImmunoResearch Laboratories Inc.), rabbit α mouse Cy3 (1:500, 315-165-044, Lot: 131676, Jackson ImmunoResearch Laboratories Inc.), Donkey α goat AF488 (1:250, 705-545-147, Lot: 136089, Jackson ImmunoResearch Laboratories Inc.), and Donkey α goat Cy3 (1:500, 705-165-147, Lot: 139052, Jackson ImmunoResearch Laboratories Inc.). 

### 2.8. Preparation of Tissue for Reverse Transcriptase-Polymerase Chain Reaction(RT-PCR) and Western Blot Analysis

For the PCR and western blot analysis, brain hemispheres were shortly thawed and Corpora callosa were dissected from the tissue with disposable scalpels (B. Braun, Melsungen, Germany). Afterward, the tissue was lysed with mRNA lysis buffer (Sigma: GenElute Mammalian Total RNA Miniprep Kit) or protein lysis buffer (50 mM Tris, 150 mM NaCl, 5 mM EDTA, 5 mM EGTA, 1% Triton X-100, 0.1% SDS, 0.1% Na-deoxycholate).

### 2.9. RT-PCR Analysis

To investigate mRNA expression in the tissue samples, mRNA was isolated from lysates and cDNA was synthesized following the manufacturer’s instructions (Sigma: GenElute Mammalian Total RNA Miniprep Kit, Thermo Fisher Scientific: First Strand cDNA Synthesis Kit). Via RT-PCR ***β-actin*** (for: TAT GCC AAC ACA GTG CTG TCT GGT GG, rev: TAG AAG CAT TTG CGG TGG ACA ATG G), ***Mbp*** (for: TCT CAG CCC TGA CTT GTT CC, rev: ATC AAC CAT CAC CTG CCT TC) and ***Pdgfrα*** (for: GCA CCA AGT CAG GTC CCA TT, rev: CTT CAC TGG TGG CAT GGT CA) were amplified. All primers were from Sigma.

### 2.10. Western Blot Analysis

Proteins were separated by weight in 12% polyacrylamide-SDS-gels and transferred after to PVDF-membranes (Carl Roth) using a semi-dry transblot system (Carl Roth). Membranes were blocked with 5% skimmed milk powder (Heirler, Radolfzell, Germany) in tris-buffered saline with Tween TBST (0.05% Tween-20, 1 × TBS; 10 × TBS: 250 mM Tris/HCl pH 7.4, 1.5 M NaCl) (blocking solution) for 1 h. Membranes were incubated in primary antibody, dissolved in blocking solution at 4 °C overnight, followed by three washing steps in TBST. Next, a one-hour incubation with the secondary antibody, which was dissolved in blocking solution, and finally, three washing steps with TBST and one wash with 1 × TBS were carried out. Western blots were developed after incubation with the substrate solution (ECL Substrate, BioRad Lab. Inc., Hercules, CA, USA) for 5 min. Antibodies: LRP1 (1:10,000, ab92544, Lot: 6R259330-27, Abcam), MBP (1:1000, MCA409S, Lot: 161031A, BioRad), PDGFRα (1:10,000, sc-338, Lot: E2015, Santa Cruz), α-tubulin (1:10,000, T9026, Lot: 078M4796 V, Sigma), Goat α rabbit HRP (1:5000, 111-035-144, Lot: 132409, Jackson ImmunoResearch Laboratories Inc.), Goat α mouse HRP (1:10,000, 115-035-068, Lot: 132223, Jackson ImmunoResearch Laboratories Inc.), and Goat α rat (1:5000, 112-035-062, Lot: 90553, Jackson ImmunoResearch Laboratories Inc.).

### 2.11. Experimental Autoimmune Encephalomyelitis (EAE)

For the analysis of the functional effects of LRP1 on oligodendrocytes, experimental allergic encephalomyelitis (EAE) was induced. Tamoxifen-treated animals were generated and immunized with MOG_35–55_ peptide (synthesized at Charité Berlin, Germany) in complete Freund’s adjuvant (incomplete Freund-adjuvant, M. tuberculosis H37 Ra, Difco Laboratories, Detroit, MI, USA) at the age of 8–10 weeks (P56–P70). Additionally, the animals received 250 ng/100 µL pertussis toxin (EMD Millipore Corporation by Merck) on the day of and two days after immunization. Clinical symptoms were evaluated using a 10-point-score scale (0 = normal, 1 = reduced tail tonus, 2 = complete tail palsy, 3 = lack of reflexive compensatory movements while walking, 4 = ataxia, 5 = slight paralysis of the hind legs, 6 = plegia of one leg or moderate paralysis of both legs, 7 = paraplegia with complete paralysis of both hind legs, 8 = tetraparesis with (slight) paralysis of front extremities, 9 = moribund, and 10 = death) and score and weight were documented on a daily basis. After 28 days of monitoring the course of disease, the experiment was stopped and the animals were sacrificed. 

### 2.12. Imaging

Immunohistochemical stains were documented with AxioZoom V16, AxioCam 506mono, and Zen 2009 software by Zeiss (Oberkochen, Germany). Three caudal and three rostral images of each Corpus callosum were taken. RT-PCR results were kept by a documentation system from LTF Labortechnik (Wasserburg, Germany) with BioCaptw software. Protein gels and western blots were imaged with the documentation system MicroChemi and Gel Capture 6.6 software by DNR bio imaging systems (Jerusalem, Israel). 

### 2.13. Quantification

Immunohistochemical stains were quantified by single cell counting in ImageJ/FIJI. Single cells were defined by nuclear stain with Hoechst dye, binding to nucleic acids. Immuno-positive cells were identified by expression of stage- or lineage-specific markers, depending on their expected localization (intranuclear, intracellular, extracellular). Three caudal and rostral sections of the Corpus callosum in the sagittal orientation were taken and at least 200–1200 cells per section were counted. 

mRNA and protein expression were measured by intensity measurements in ImageJ/FIJI. 

In some cases, significant reductions between two conditions were additionally calculated and mentioned in the text. Therefore, the higher value served as 100%. 

### 2.14. Statistics

Statistics were depicted as mean ± SEM. Normal distribution of values was checked with the Shapiro–Wilk test. Depending on normally or not normally distributed values, the Student’s *t*-test or Mann–Whitney U test was performed. Biological replicates were stated as “N” and technical replicates as “n”. Immunohistochemical stains: N = 3–4, n = 9–12; PCR analysis: N = 4, n = 4; western blot analysis: N = 3, n = 3; EAE pilot study: N = 5, n = 5. Significant data were declared with a *p*-value (*p*) of 0.05. *p* ≤ 0.05 for *, *p* ≤ 0.01 for ** and *p* < 0.001 for ***. All statistical tests were performed with Microsoft Excel (Redmond, WA, USA). 

## 3. Results

### 3.1. Induction of the NG2-Restricted LRP1-Deficient KO in the New Mouse Model

In order to investigate the role of LRP1 in the NG2-cell lineage, we generated the novel conditional knockout mouse model: NG2-CreERT2^ct2/wt^xR26eGFP^flox/flox^xLRP1^flox/flox^ (KO) and NG2-CreERT2^wt/wt^xR26eGFP^flox/flox^xLRP1^flox/flox^ (control). To induce the deletion, tamoxifen was applied to lactating mothers three and four days after the birth of the litters. For the analysis, we focused on six different age stages to evaluate different experimental aspects (Figure 1A). 

First, we focused on the number of cells that recombined upon tamoxifen treatment by monitoring the expression of the reporter GCaMP3 detectable by anti-GFP antibodies. For the sake of simplicity, we refer to GCamP3-expressing cells as GFP- or reporter-expressing cells. Cells were counted in the Corpus callosum because this brain region represents a strongly myelinated fiber tract. Overall cell numbers were visualized using a nuclear marker (Hoechst dye) and the proportion of immunostained cells was recorded. Postnatally from P7 to P42, an increase in the number of GFP-expressing cells could be observed, before the fraction of immunopositive cells dropped rapidly at P56 (Figure 1B,C). For the rostral Corpus callosum, a range of maximally 31% ± 4.9% standard error of the mean (SEM) and minimally 7% ± 1.6% SEM was found over time. Similar to this finding, a maximum of 27% ± 9% SEM and a minimum of 4.7% ± 1.4% GFP-positive cells was present in the caudal region of the Corpus callosum (Figure 1C). These results suggest that initially from P7 to P42, about one third of the cells in the Corpus callosum showed Cre-mediated recombination, which represents the percentage of oligodendrocyte lineage cells generated by NG2 cells present at P3 to P5 at the time of tamoxifen injection.

To further assess the number of recombined cells that could be attributed to the oligodendrocyte lineage, we employed the lineage-specific marker Olig2 and analyzed the number of cells double-positive for GFP and Olig2. At P7, half of all oligodendrocytic Olig2-positive cells expressed GFP and revealed a recombination efficiency of 50% recombined LRP1-deficient oligodendrocytes. Surprisingly, we saw a significant reduction in the number of recombined oligodendrocytes over time (Figure 1D,E). Focusing at the maximum at P7, fractions of 53% ± 9.9% SEM rostrally and 59% ± 6.7% SEM caudally of GFP-expressing Olig2-positive cells were identified for the Corpus callosum. Reflecting a loss of recombined oligodendrocytes, a minimum of only 15% ± 6.6% SEM (rostral) and 13% ± 2.8% SEM (caudal) of GFP-expressing Olig2-positive cells was left at P56. Setting these fractions in relation to the starting point at P7, this signifies a rostral and caudal reduction of recombined oligodendrocyte lineage cells by 71.1% (53% ± 9.9% SEM reduced to 15% ± 6.6% SEM) and 77.3% (59% ± 6.7% SEM reduced to 13% ± 2.8% SEM), respectively. This strong effect illustrated a clear linkage between LRP1 expression and oligodendrocyte lineage cell survival. The most straightforward explanation for the progressive loss of recombined cells in the Olig2-lineage is presumably cell death as a consequence of LRP1 elimination.

### 3.2. LRP1 Expression in Oligodendrocyte Development

**Figure 2:**
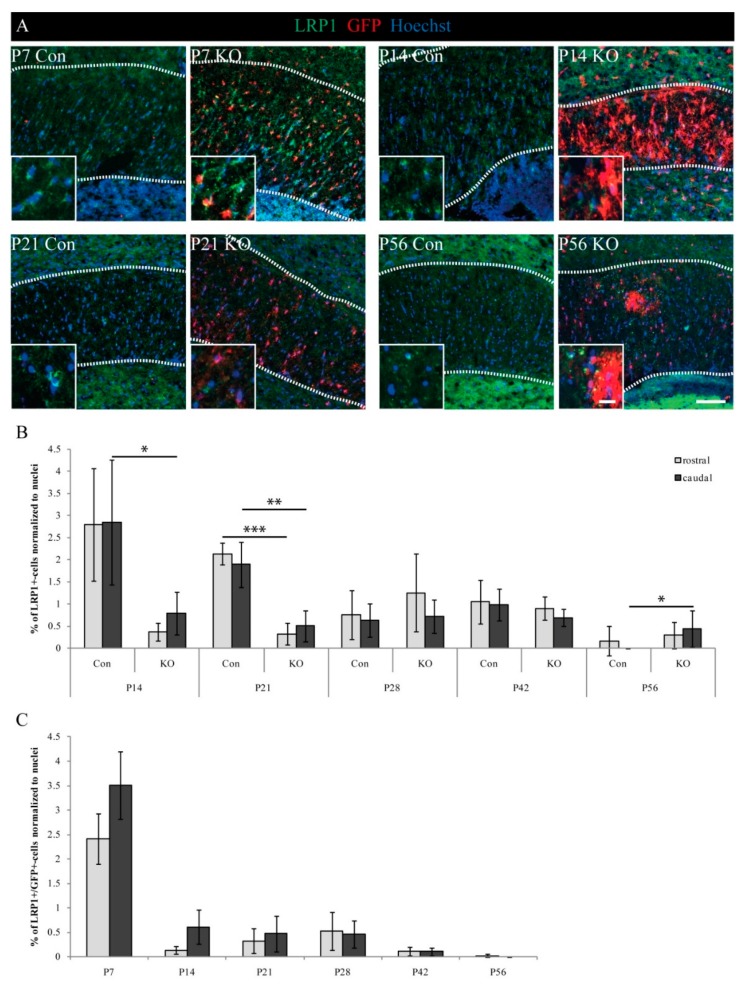
LRP1-expressing cells decreased over time. (**A**) Immunohistochemical staining against LRP1 and GFP to identify LRP1-expressing cells and double-positive cells, indicating reporter-positive cells that still expressed non-degraded LRP1 (scale bars: 100 µm, 20 µm). (**B**) Quantification of LRP1+-cells indicated a time-dependent reduction and proved the success of the inducible KO strategy with significantly reduced numbers of LRP1-expressing cells in the KO at P14 and P21 (* *p* ≤ 0.05; ** *p* ≤ 0.01; *** *p* ≤ 0.001). (**C**) Time course of cell loss with LRP1 expression. Depicted is the remaining fraction of cells that were still double-positive for LRP1 and GFP. Please note that different experimental age stages are shown in diagrams (**B**,**C**). Data are expressed as the mean ± SEM. N = 3–4, n = 9–12 per rostral and caudal part. At least 200–1200 cells per section were counted. Depending on normally or not normally distributed data, the Student’s *t*-test or Mann–Whitney U test was used for evaluation within the individual ages.

The new mouse model aims at LRP1-deficiency in OPCs and differentiating oligodendrocytes. Therefore, the analysis of LRP1-expressing cells was vitally important for the characterization of the model and the impact of LRP1 on oligodendrocytes. So, we performed immunohistochemical staining against LRP1 in relation to all cells assessed by nuclear stain (Figure 2 and Figure S2). Previous studies have demonstrated a decrease in LRP1-expression on the mRNA and protein level in the oligodendrocyte lineage during development [9,12], which was verified in our model during development with LRP1-expressing cells. LRP1-positive cells declined from P7 (Con maximum: 9.0% ± 1.9% SEM) to P56 (Con maximum: 0.2% ± 0.3% SEM) (Figure 2A,B). To assess the success of LRP1 deletion on protein level immunohistochemical staining against LRP1 were performed. Here, comparable fractions of LRP1-positive cells were observed in the KO when compared to the control at P7 (Con maximum: 9.0% ± 1.9%; KO maximum: 7.6 ± 2.0%). This indicates an incomplete recombination, or rather incomplete degradation of LRP1, at this time point shortly after tamoxifen-mediated induction. Notable differences were observed at P14 and P21, with significantly reduced fractions of LRP1-expressing cells in the KO when compared to the control. This demonstrated a successful recombination in terms of LRP1 deletion and the degradation of LRP1. In detail, we found a significant reduction by 72% (2.8% ± 1.4% SEM reduced to 0.8% ± 0.5% SEM) of LRP1-expressing cells from the control to KO condition at P14 in the caudal Corpus callosum (*p* ≤ 0.05, *). One week later, highly significant reductions by 85% (2.1% ± 0.2% SEM reduced to 0.3% ± 0.3% SEM) rostrally and 73% (1.9% ± 0.5% SEM reduced to 0.5% ± 0.4% SEM) caudally in comparison with the control were shown for the KO at P21 (*p* ≤ 0.01 **; *p* ≤ 0.001 ***). Thus, a KO-dependent decrease in the LRP1-expressing cells was observed that confirmed the success of the inducible KO strategy. Accordingly, the loss of LRP1 in these cells might account for the previously detected reduced proportion of recombined cells (see above). 

Strikingly, a significant difference between the control and KO was observed at P56. Whereas no LRP1-positive cells were found in the caudal Corpus callosum of the control, 0.5% of the cells expressed LRP1 in the KO (* *p* ≤ 0.05). 

In a complementary approach, we analyzed the time-dependent change of the cell fraction still double-positive for GFP and LRP1. This fraction represents the surviving cells of the oligodendrocyte lineage due to still incomplete protein degradation [23]. Immunohistochemical staining against LRP1 and GFP revealed a maximum at P7 with 2.4% ± 0.5% SEM (rostral) and 3.5% ± 0.7% SEM (caudal) of LRP1- and GFP-double-expressing cells. Within the next seven days, the fraction of double-positive cells dropped rapidly to 0.5% and less, and at P56, a minimum of 0.02% ± 0.03% SEM (rostral) and 0% (caudal) remained (Figure 2A,C). In conclusion, more than 95% of cells with recombined, and therefore activated reporter were devoid of LRP1 protein.

### 3.3. Proportion and Distribution of Control and KO Cells

**Figure 3:**
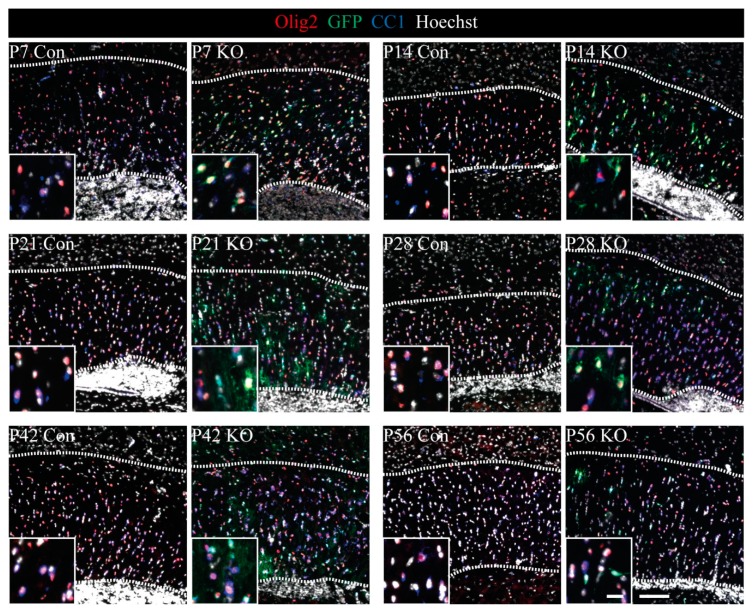
Triple immunohistochemical stainings showing mature and immature proportions within oligodendrocyte lineage in the Corpus callosum by Olig2, CC1, and GFP. Immunohistochemical stainings of P7–P56 control and KO tissue against Olig2, GFP, and CC1 (mature con cells defined as: Olig2+/CC1+/GFP−; mature KO cells: Olig2+/CC1+/GFP+; immature con cells defined as: Olig2+/CC1−/GFP−; immature KO cells: Olig2+/CC1−/GFP+) (scale bars: 100 µm, 20 µm).

In order to investigate the LRP1 KO-dependent effects specifically in the oligodendrocyte lineage, we analyzed the proportion and distribution of immature and mature oligodendrocytes in vivo. To this end, we prepared triple immunostainings to define different subpopulations of oligodendrocytes (Figure 3). 

**Figure 4:**
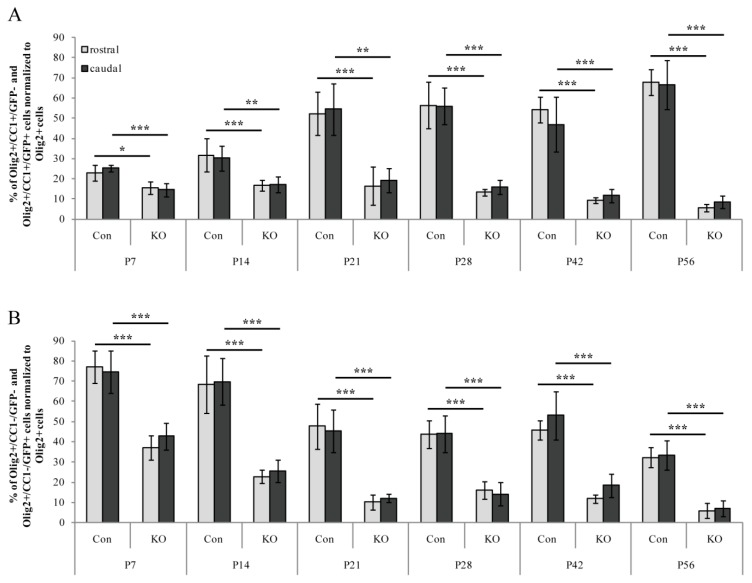
Comparison of mature and immature oligodendrocytes revealed significantly decreased fractions in the KO when compared to the control, indicating a progressive cell loss. (**A**) Quantification of mature proportions of recombined (Olig2+/CC1+/GFP+) and non-recombined oligodendrocytes (Olig2+/CC1+/GFP−), normalized to Olig2-positive cells indicated a significantly impaired KO fraction compared to the control (* *p* ≤ 0.05; ** *p* ≤ 0.01; *** *p* ≤ 0.001). This observation demonstrated a progressive loss of LRP1-deficient oligodendrocytes during development. (**B**) Quantification of immature proportions of recombined (Olig2+/CC1−/GFP+) and non-recombined oligodendrocytes (Olig2+/CC1−/GFP−), normalized to Olig2-positive cells showed comparable results to the mature proportions. A significantly reduced fraction of LRP1-deficient oligodendrocytes developed over time (*** *p* ≤ 0.001). Data are expressed as mean ± SEM. N = 3–4, n = 9–12 per rostral and caudal part. At least 200–1200 cells per section were counted. Depending on normally or not normally distributed data, the Student’s *t*-test or Mann–Whitney U test was used for evaluation within the individual ages.

**Figure 5:**
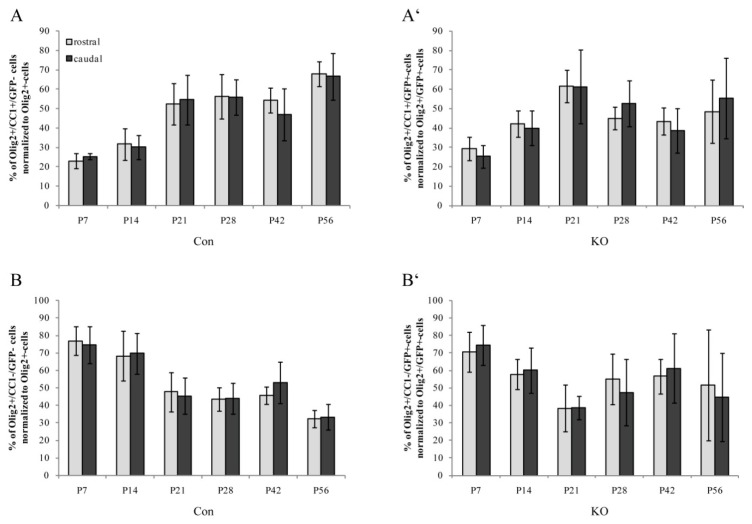
Proportions of persisting mature and immature oligodendrocyte lineage cells revealed similar differentiation and distribution in the KO condition when compared to the control. (**A**) Quantification of mature control oligodendrocyte proportions (Olig2+/CC1+/GFP− cells) normalized to Olig2+-cells revealed an increase from 20% to 65% over time. (**A’**) Quantification of mature KO oligodendrocyte proportions (Olig2+/CC1+/GFP+-cells) normalized to recombined Olig2+/GFP+-cells demonstrated an increase from P7 to P21 and comparable cell proportions afterwards. (**B**) Quantification of immature control oligodendrocyte proportions (Olig2+/CC1−/GFP-cells) normalized to Olig2+-cells exhibited decreasing proportions from 80% to 30% during development. (**B’**) Quantification of immature KO oligodendrocyte proportions (Olig2+/CC1−/GFP+-cells) normalized to Olig2+/GFP+-cells indicated a slight decrease over time. Please note that the presented proportions of mature and immature KO cells refer to the progressively decreasing recombined KO fraction (see Figure 1E). The minority of surviving LRP1-negative oligodendrocytes behaved similarly to the control. No statistically significant differences were observed between the KO and control conditions. Data are expressed as the mean ± SEM. N = 3–4, n = 9–12 per rostral and caudal part. At least 200–1200 cells per section were counted. Depending on normally or not normally distributed data, the Student’s *t*-test or Mann–Whitney U test were used for evaluation within the individual ages.

Olig2 was used to label all oligodendrocytic cells. CC1-expressing cells were defined as mature oligodendrocytes, whereas CC1-negative cells were considered as immature cells. Based on the expression of GFP, KO cells (recombined) could be discriminated from non-recombined cells within the KO tissue. In this approach, mature oligodendrocytes were visualized by the marker combinations Olig2+/CC1+/GFP− (control; non-recombined in control tissue) and Olig2+/CC1+/GFP+ (KO; recombined in KO tissue). Immature oligodendrocytes were identified as Olig2+/CC1−/GFP− (control; non-recombined in control tissue) and Olig2+/CC1−/GFP+ (KO; recombined in KO tissue) (Figure 3, Figure 4 and Figure 5).

First, the total numbers of Olig2-expressing cells served as references (100% value for the control and KO) for the analysis of mature and immature oligodendrocyte proportions in the control and KO tissue (Figure 4 and Figure 5). For mature oligodendrocytes, an increase from P7 to P56 was observed in the control animals (rostral: 23.0% ± 3.9% to 67.8% ± 6.4%; caudal: 25.4% ± 1.7% to 66.6% ± 12.0%). However, the KO fraction supported our previously found progressive loss of LRP1-deficient oligodendrocytes with decreasing proportions in mature cells during development (rostral: 15.5% ± 3.2% to 5.7 ± 1.9%; caudal: 14.6% ± 3.3% to 8.6% ± 3.2%). For this comparison a highly significant impairment of the KO condition was observed when compared to the control (* *p* ≤ 0.05; ** *p* ≤ 0.01; *** *p* ≤ 0.001). 

Furthermore, immature oligodendrocytes were investigated from P7 to P56 and revealed decreasing cell proportions for the control condition (rostral: 77.0% ± 8.0% to 32.2% ± 4.9%; caudal: 74.6% ± 10.5% to 33.4% ± 7.4%). Similar to mature LRP1-deficient KO oligodendrocytes, the proportion of immature KO cells also decreased over time (rostral: 37.2% ± 6.1% to 6.0% ± 3.7%; caudal: 42.8% ± 6.5% to 6.9% ± 3.9%). Again, a highly significant difference was seen between the control and the KO condition (*** *p* ≤ 0.001). In summary, these findings support our previously shown data (Figure 1) regarding the progressive loss of LRP1-deficient oligodendrocytes compared to the control condition and the LRP1-dependent survival of oligodendrocytes. 

In a further step, Olig2- (in the control tissue) as well as Olig2- and GFP-double expression (recombined cells in the KO tissue) were used to label the whole oligodendrocyte lineage fractions (100% reference values for the control and KO, respectively) (Figure 3 and Figure 5). For this aspect, it is important to note that the following data showed relative proportions of mature/immature cells within the recombined LRP1-free cell fraction compared to the control. As shown in Figure 1 and Figure 4, this recombined cell fraction was strongly reduced over time (compare Figure 1 reduction rostral: 71.1%, value: 53% ± 9.9% SEM reduced to 15% ± 6.6% SEM; reduction caudal: 77.3%, value: 59% ± 6.7% SEM reduced to 13% ± 2.8% SEM; see also Figure 4 and description above). This means that the absolute numbers of such KO cells found in the Corpus callosum were much lower than the control.

Focusing on the proportion of mature cells, a non-linear increase over time was observed (minimum: 23.0% ± 3.9% SEM, maximum: 67.8% ± 6.4% SEM) (Figure 5A,A’). At P21, the first peak was reached and the maximum of KO oligodendrocytes (67.8% ± 6.4% SEM) was detected, which slightly decreased until P56 (minimum 48.5% ± 16.4% SEM) (Figure 5A’). In contrast, the control condition revealed the opposite effect with a maximum at P56 (P21: 52.3% ± 10.6% SEM; P56: 67.8% ± 6.4% SEM) (Figure 5A). 

The proportion of immature cells tended to decrease (Figure 5B,B’). From P7 to P21, a clear reduction in the fraction of immature cells was observed (maximum: 77.0% ± 8.0% SEM, minimum: 38.3% ± 13.3% SEM), which was comparable in both genotypes. Thereafter, the control cell fraction remained stable until P42 and finally decreased until P56 (minimum: 32.2% ± 4.9% SEM) (Figure 5B). Focusing on the KO, an increase to P42 (maximum: 61.3% ± 19.7% SEM) was determined, followed by a slight decrease until P56 (minimum: 44.7% ± 25.1% SEM) (Figure 5B’). 

Overall, the distribution of mature and immature oligodendrocytes in both genotypes was comparable at the individual postnatal age stages. However, the proportion of immature KO cells increased after P21, whereas the relating ratio of mature cells decreased in this LRP1-deficient fraction. In fact, our data suggest that although progressive elimination of Olig2-positive cells without LRP1 expression was observed via the loss of Olig2/GFP-double positive cells (Figure 1 and Figure 4), the remaining fractions of oligodendrocyte lineage cells within the Corpus callosum revealed comparable characteristics. We assumed a compensatory effect by regenerative healthy cells at regions of progressive cell loss. The smaller proportion of remaining, recombined KO cells behaved similarly with regard to distribution and differentiation in the Corpus callosum compared to the control cells. 

### 3.4. Cellular Characterization of Oligodendrocyte Lineage-Specific Cells

**Figure 6:**
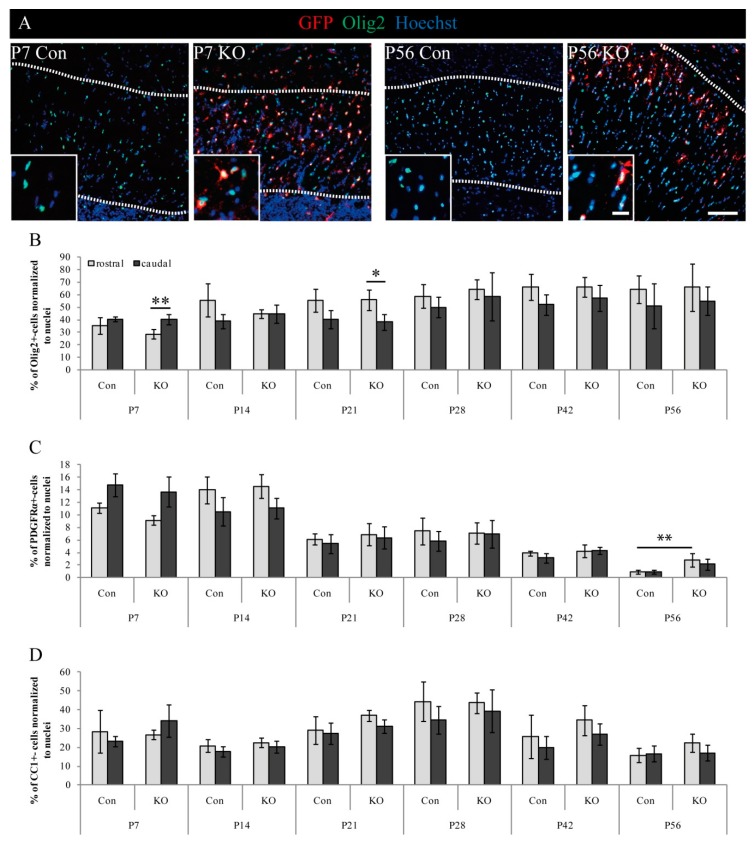
Analysis of stage-specific oligodendrocyte markers. (**A**) Representative immunohistochemical stainings against Olig2 and GFP for the control and KO animals at P7 and P56 (scale bars: 100 µm, 20 µm). (**B**) Quantification of Olig2+-cells indicated a slight increase over time with similar results for both genotypes. Significant caudo-rostral differences were observed in the KO at P7 and P21 (* *p* ≤ 0.05; ** *p* ≤ 0.01). (**C**) Quantification of PDGFRα+-OPCs from P7 to P56, demonstrating globally decreasing cell numbers and a significantly higher proportion of precursors in the KO when compared to the control at P56 (** *p* ≤ 0.01). (**D**) Quantification of CC1-expressing mature oligodendrocytes revealed increasing numbers up to P28, followed by a decrease afterward. No difference was detectable between the control and KO. Data are expressed as mean ± SEM. N = 3–4, n = 9–12 per rostral and caudal part. At least 200–1200 cells per section were counted. Depending on normally or not normally distributed data, the Student’s *t*-test or Mann–Whitney U test were used for evaluation within the individual ages (see supplementary analysis in Figure S3).

After the evaluation of the KO and successful recombination, we wanted to exclude side-effects on the oligodendrocyte population due to the tamoxifen-induced recombination. We aimed to compare oligodendrocyte lineage cell fractions in the Corpus callosum in a genotype-independent manner (including GFP+ and GFP− cells in the tissue) to validate potential gross differences in tissue composition between the control and KO animals. Therefore, subpopulations of oligodendrocytes of various differentiation stages (e.g., precursors and mature cells) were investigated. To this end, immunohistochemical stainings, using the lineage marker Olig2 (including OPCs and mature oligodendrocytes), the OPC marker PDGFRα (only OPCs), and the mature marker CC1 (mature oligodendrocytes only) were performed (Figure 6). Proportions of labeled cells were determined in relation to the overall cell number as revealed by staining the cell nuclei (Figure 6; see alternative analysis in Figure S3). 

Focusing on the total number of Olig2-positive cells, we found slightly increasing numbers over time in both genotypes, starting with a minimum of 28.6% ± 3.8% SEM in the rostral KO Corpus callosum at P7, and reaching a maximum fraction of 65.9% ± 19.1% SEM in the rostral part at P56 (Figure 6A,B). Furthermore at P7 and P21, the KO conditions showed significantly different distributions of Olig2-positive cells within the Corpus callosum (* *p* ≤ 0.05; ** *p* ≤ 0.01). These can be explained with rostro-caudal differences due to differentiation, myelination, and development, which were the reason for the region-specific analysis in the present study [24,25,26,27,28]. As expected, the number of oligodendrocyte lineage cells in the Corpus callosum augmented with time. 

Proceeding more specifically with progenitor and mature oligodendrocyte fractions, we noted inverse effects (Figure 6C,D). PDGFRα-positive OPCs decreased over time in the control and KO, starting with 17.8% ± 1.9% SEM (maximum, caudal, control) at P7, whereas only 0.9% ± 0.3% SEM (minimum, caudal, control) immunopositive cells remained in the adult stage at P56 (Figure 6C). In addition, a significantly increased OPC-fraction was observed at P56 for the KO in the rostral Corpus callosum compared to the control (** *p* ≤ 0.01). In contrast, CC1-positive mature oligodendrocytes tended to increase within the first four weeks (until P28) (increase: 15%; value: 23.1% ± 2.7% SEM up to 44.3% ± 10.4% SEM) and surprisingly decreased afterward (reduction: 28%; value: 44.3 % ± 10.4% SEM reduced to 15.9% ± 3.8% SEM) (Figure 6D). Thus, the OPC population progressively matured, losing the PDGFRα marker, and expressing CC1. These data support the interpretation that the progressive loss of recombined LRP1-deleted oligodendrocytes is compensated by non-affected cells.

### 3.5. Molecular Characterization of Oligodendrocyte Lineage-Specific Cells

**Figure 7:**
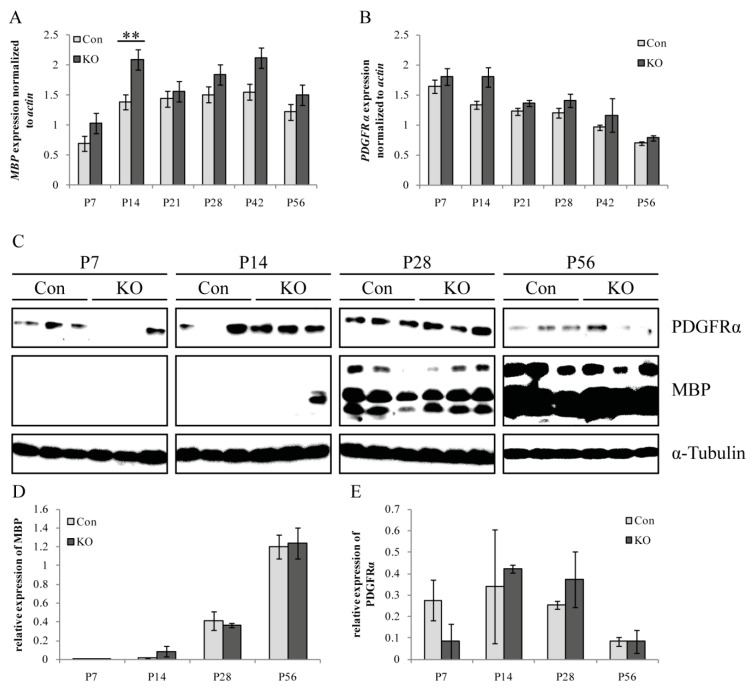
mRNA and protein levels of oligodendrocyte-specific markers were not impaired due to loss of LRP1. (**A**) Quantification of the relative expression of *Mbp* mRNA revealed a time-dependent upregulation with a significantly higher expression in the KO at P14 (** *p* ≤ 0.01). (**B**) Quantification of the relative expression of *Pdgfrα* mRNA showed a downregulation from P7 to P56 in the control and KO. (**C**) Western blot analysis of protein levels of PDGFRα and MBP. α-tubulin served as the loading control. (**D**) Quantification of the relative expression of MBP on protein level demonstrated a strong time-dependent upregulation of about 100% from P7 to P56. (**E**) Quantification of the relative expression of PDGFRα protein level illustrated a tendential downregulation from P14 to P56. Data are expressed as the mean ± SEM. PCR: N = 4, n = 4, western blot: N = 3, n = 3. Depending on normally or not normally distributed data, the Student’s *t*-test or Mann–Whitney U test was used for evaluation within the individual ages.

The above-mentioned results of the cellular characterization of oligodendrocytic cells by immunohistology were complemented by RT-PCR and western blot analysis referring to PDGFRα and MBP (myelin basic protein, a mature myelin marker) expression on the mRNA and protein level. Corpora callosa of the control and KO animals were prepared (Figure 7). In agreement with the results of immunohistochemistry, the RT-PCR and western blot results demonstrated increasing levels of the mature marker MBP and decreasing levels of the OPC marker PDGFRα over time (Figure 7A–E). Overall PCR and western blot analysis generally confirmed the previous findings obtained by immunohistochemistry, where a comparable outcome of oligodendrocyte maturation in both genotypes was observed. However, as an exception, we saw a significantly upregulated relative *Mbp* mRNA expression in the KO when compared to the control at P14 (** *p* ≤ 0.01). 

In summary, the immunohistochemistry, RT-PCR, and western blot results suggested comparable rates of oligodendrocyte differentiation in response to the tamoxifen treatment excluding strong unspecific recombination-induced side-effects in our experimental approach, which could interfere with our analysis of cell-autonomous effects. 

### 3.6. Experimental Autoimmune Encephalomyelitis (EAE)

**Figure 8:**
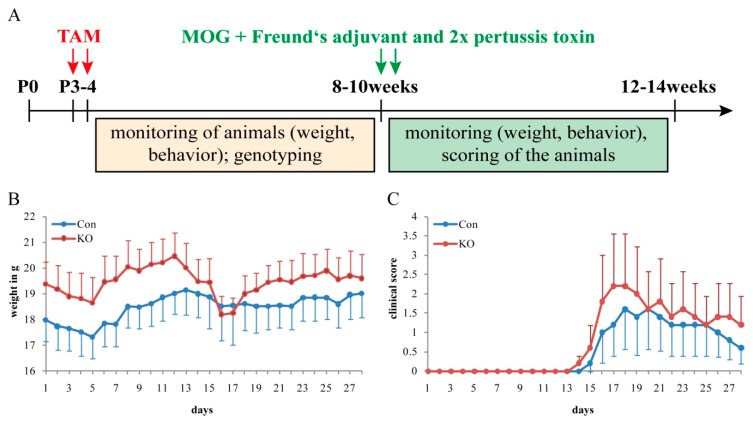
Experimental allergic encephalomyelitis (EAE) immunization reveals first hints toward more strongly affected KO animals with greater deficits when compared to the control in the proof of concept experiment. (**A**) Timeline of experimental procedure. (**B**) Diagram of documented weights (g) during the course of disease over four weeks of control and KO animals. (**C**) Diagram of relating clinical scores of the control and KO animals over four weeks of EAE. Data are expressed as the mean ± SEM. N = 5, n = 5. Depending on normally or not normally distributed data, the Student’s *t*-test or Mann–Whitney U test was used for evaluation within the individual ages. Score: 0 = normal; 1 = reduced tail tonus; 2 = complete tail palsy; 3 = lack of reflexive compensatory movements while walking; 4 = ataxia.

Oligodendrocytes form myelin sheaths indispensable for integral CNS function and that are damaged in neuroinflammatory diseases. We wanted to examine whether LRP1 deletion compromises the myelination by oligodendrocytes. From this perspective, we induced EAE in tamoxifen-treated animals of both genotypes and sexes at the age of 8 to 10 weeks (P56–P70) and monitored and scored the animals for four weeks (Figure 8). The two parameters investigated in this study were the body weight and the clinical score of the animals over four weeks of the disease course (Figure 8B,C). Focusing on weight first, KO animals started with a slightly higher weight of 19.4 g ± 0.8 g SEM when compared to the control with 18 g ± 1.8 g SEM. 

Within the first five days post immunization (dpi), a slight decrease of body weight was observed in both genotypes (control: 17.3 g ± 1.8 g SEM; KO: 18.7 g ± 0.8 g SEM), followed by an increase until 12 or rather 13 dpi, respectively (control: 19.1 g ± 2.1 g SEM; KO: 20.4 g ± 0.8 g SEM) (Figure 8B). While animals of the control condition remained constant in body weight until the end of the study (around 18.5–19 g), the KO animals demonstrated a notable reduction in body weight after 12 dpi with a minimum of 18.2 g ± 1.32 g SEM at 16 and 17 dpi.

The individual KO animals in our study appeared to be clearly affected by the EAE immunization, as indicated by the diminution in body weight. Following this reduction, the body weight of the KO animals increased again (up to 22 dpi), transiting into a stable phase until the end of the study (Figure 8C). 

According to the clinical score, as an indicator for the severity of symptoms due to EAE immunization (Figure 8C), both genotypes responded equally at the beginning of the experiment. A score of 0 revealed a lack of symptoms up to 13 dpi. Afterward, animals of both genotypes developed symptoms, reflected by an increased score of 1.6 in the control and 2.2 in the KO (control: 18 and 20 dpi, KO: 17 and 18 dpi). This score reflected a combination of a reduced tail tonus and complete tail palsy in control animals versus complete tail palsy in conjunction with a lack of reflexive compensatory movements while walking in KO animals. The proceeding disease course demonstrated a remitting phase in both genotypes with decreased scoring until the end of the experiment. In the control, a score of 0.6 indicated an intermediate stage between normal behavior and reduced tail tonus, whereas the KO condition was still more strongly affected with a score of 1.2 (Figure 8C). It has to be noted that our study comprised small collectives revealing mild differences in body weight and clinical score that did not, however, achieve statistical significance. This may be due to the fact that the majority of LRP1-deleted OPCs has been eliminated from the CNS of triple transgenic mice, as described above. Future studies will have to be performed to verify the asserted involvement of LRP1 in the recovery from EAE. 

## 4. Discussion

In order to study LRP1 in the oligodendrocyte lineage, we decided to generate a tamoxifen-inducible conditional mouse line as it enables the selection of defined induction time points for the deletion of the gene. With our induction protocol, we benefited from the NG2-CreERT2 mouse, which was used to generate our novel model [21]. Depending on the reporter genes eYFP and tdTomato, recombination efficiencies of 50–95% respectively were observed by Huang et al. [21] and corresponded to our data at P7.

In our study, we present a new triple transgenic mouse model with LRP1-deficiency induced in postnatal OPCs: NG2-CreERT2^ct2/wt^xR26eGFP^flox/flox^xLRP1^flox/flox^ (KO). Deletion of LRP1 in postnatal OPCs and their progeny resulted in the progressive loss of KO oligodendrocytes during development. Furthermore, EAE immunization hints at a clear response in LRP1 KO animals. On the cellular level, an in vitro myelination assay provided initial evidence for modified myelination behavior of recombined oligodendrocytes, as indicated by elongated internodes in the LRP1 KO condition (Figure S1). Focusing on the oligodendrocyte lineage, we were able to uncover a new role of LRP1 in oligodendrocyte survival during development in vivo and potentially also myelin formation in vitro. 

In order to target the oligodendrocyte lineage, we induced the KO at P3 and P4 by injection of tamoxifen to lactating mothers, which metabolized tamoxifen to 4-hydroxy-tamoxifen in the liver [29] and fed the metabolites to the pups by milk. Early oligodendrocyte development is characterized by three independent OPC waves, which populate the forebrain at embryonic day (E) 11.5, E15.5, and P0, and are derived from different sources or structures of the early brain [15,30,31]. Based on the induction of the KO in young postnatal animals, we wanted to guarantee the deletion of LRP1 from a huge proportion of OPCs in their target compartment and aimed to analyze the effect of the lineage-restricted LRP1-deficiency in oligodendrocytes over time. 

Additional to our focus on the oligodendrocyte lineage, the new mouse model also allowed us to investigate LRP1-deletion from NG2-glia with an adapted injection protocol (the tamoxifen treatment can be adjusted depending on the experimental question). The proteoglycan NG2 is not only expressed in OPCs [18,19], but also in pericytes, which are associated with blood vessels [32]. Pericytes are known to express LRP1 and therefore could have been affected in our conditional knockout mouse model. However, our experimental focus was on oligodendrocytes and myelination. Therefore, we used lineage-specific markers such as PDGFRα, CC1, MBP, and Olig2 to address this aspect in immunohistochemical stainings. PCR and western blot analysis were used to confirm the effects of LRP1 deletion on cells of the oligodendrocyte lineage. Additionally, for the in vitro myelination assay pure OPC cultures were used to compare LRP1-deficient OPCs with control cells. At present, we cannot exclude a potential intervention of LRP1-deficient pericytes in the EAE experiment. 

OPCs not only exist in the young brain where they differentiate to oligodendrocytes, but proliferate and can be found throughout the brain into adulthood and were recently termed NG2 glia [33]. These macroglia-like cells in the CNS can generate oligodendrocytes and astrocytes during development [31,34]. In addition, NG2 glia has been suggested as an immature, progenitor-like cell type that can differentiate into mature cells with neural properties depending on specific environmental stimuli [35]. They are present in white and grey matter, but have also been found in stem cell niches such as the subventricular zone (SVZ) [33,36,37]. So far, nothing is known about LRP1 protein expression in NG2 glia, but the precursor properties of NG2 glia for oligodendrocyte lineage suggest an upregulation of LRP1 in NG2 glia. Our triple transgenic mouse model offers new possibilities to investigate LRP1-deletion in NG2 glia at different deliberately chosen time points. LRP1 deficiency from neural cell types results in multiple cellular or functional disorders (e.g., apoptosis in neurons [4], synapse loss and neurodegeneration in aging mice [38], and deficits in chemically induced white matter lesion repair [16]). Based on the knowledge concerning NG2 glia and LRP1, we propose potential effects of LRP1 on NG2 glia and their functions. 

Deletion of LRP1 in postnatal OPCs in the new mouse model demonstrated a significant reduction of recombined cells during development. This indicated a progressive loss of LRP1-deficient oligodendrocytes in the Corpus callosum, the structure with the highest density of oligodendrocytes [39,40]. From this, we concluded a vulnerable phenotype of LRP1-deficient oligodendrocytes when compared to the control cells. To examine a potential global reduction of oligodendrocyte numbers during development due to the progressive loss of KO oligodendrocytes, we visualized the total fraction of oligodendrocytes with the help of Olig2, a specific lineage marker [41,42]. Increasing numbers of oligodendrocytes in the Corpus callosum over time support our interpretation that the progressively lost LRP1-deficient oligodendrocytes are replaced by GFP-negative non-recombined control cells in the KO over time. 

Nevertheless, what happens to the disappearing, presumably weak and vulnerable LRP1 KO oligodendrocytes? We have previously shown that LRP1-deficient neural stem cells exhibited higher apoptosis rates when compared to the control conditions [11]. Pro-apoptotic effects in the absence of LRP1 have also been reported in neurons [4]. From this, we can conclude that the loss of LRP1-deficient oligodendrocytes could be explained by cell death, presumably apoptosis. 

In order to study potential roles in myelination-related diseases, MOG_35–55_ EAE was induced in the control and KO animals. EAE is a model that mimics multiple sclerosis pathophysiology with focal inflammatory, demyelinating lesions, and axonal damage [43,44]. Furthermore, MOG_35–55_ EAE immunization is expected to generate a monophasic disease course [45], as also confirmed with our clinical score data. Defects preferentially appear in the spinal cord, but might also occur in the cerebellum and optic nerve, whereas symptoms are absent from the forebrain (including cortex and Corpus callosum) [43,44,45]. In explaining our preliminary findings in affected KO animals, the influences regarding the immune system should also be considered as the EAE model mimics autoimmune disease. Previously, anti-inflammatory effects of LRP1 in lipopolysaccharide, tumor necrosis factor α, and growth factor signaling as well as in phagocytosis have been observed [46]. These reports suggest an important role of LRP1 in the immune system. On the other hand, the LRP1 deletion in our model is restricted to the NG2-dependent lineage, and therefore it is questionable whether the immune system is modified in the EAE study. Furthermore, our observation of vulnerability in LRP1-deficient KO cells provides the first evidence for impairment in oligodendrocytes and potentially in myelin in our novel triple transgenic mouse model. Although these data refer to findings in the brain (Corpus callosum) and not the spinal cord, we expect similar conditions regarding cell fractions and recombination efficiency in the caudal sections of the CNS. We assumed that the affected KO animals might point to a late effect of progressive loss of LRP1-deficient oligodendrocytes. It is imaginable that the regenerative capacity of healthy LRP1-expressing oligodendrocytes is impaired at regions of progressive loss of LRP1 KO cells. As a consequence, vulnerable myelin might be formed around those axons exposed from myelin sheaths of perished oligodendrocytes, which might appear under EAE disease conditions. This concept will have to be validated by studies designed to eliminate LRP1 from non-recombined cells in parallel to EAE initiation. Our triple transgenic mouse model offers the option of timed elimination of LRP1 from OPCs recruited for myelin regeneration.

Based on our assumptions of altered myelin in the KO condition, we performed an in vitro myelination assay. Here, differentiated OPCs were placed on artificial fibers to investigate potential myelin impairment (Figure S1). This method allows the lengths of myelin sheaths, or rather internodes, to be measured [47]. Thereby it enables—and already has been used—to identify alterations in different conditions (e.g., due to genetically modified OPCs [48]). This myelination assay, performed with cortex-derived OPCs, revealed seemingly elongated internodes in the KO condition when compared to the control. Correlated with our findings of EAE, this in vitro study might point to myelin impairment in NG2-restricted LRP1-deficient mice in vivo. Again, one has to keep in mind that OPCs were derived from brain structures, and not from spinal cord, although this impaired myelination might represent a confident target of EAE inflammation. The elongated internodes might also display myelin dysregulation, referring to impaired thickness and/or wrapping of myelin, which could be addressed by electron microscopy. 

The myelination assay suffered from strongly reduced numbers of surviving recombined KO cells in the immunopanning procedure and cultivation of cells on fibers. This underlined the weak and vulnerable KO cell phenotype in vitro and is in agreement with our previous observations. To circumvent the problems of OPC cultivation, in a future perspective, cells from another mouse model could be used that shows a higher proportion of recombined (KO) cells. 

In addition to our observations regarding LRP1 loss and its marked consequences in the oligodendrocyte lineage, we also analyzed parameters that revealed a rather mild outcome. Namely, we concentrated on immature and mature oligodendrocytes in a global (control tissue vs. KO tissue including recombined and non-recombined cells) and cell-specific manner (control cells in control tissue vs. recombined cells in KO tissue). Decreasing proportions of precursors and increasing proportions of mature cells indicated oligodendrocyte differentiation and maturation over time [15,49,50]. mRNA and protein levels of PDGFRα (precursor marker) and MBP (mature marker) confirmed findings obtained by immunohistochemistry. Moreover, we found normally behaving and developing KO animals when compared to the control litter. 

Despite the normal appearance of the mice, we found an important role of LRP1 in oligodendrocyte survival and vulnerability, which suggests a mechanism of compensation in our new model. The first plausible mechanism relies on the observed replacement of weak and apoptotic cells by healthy control cells, which might provide an immediate, but not optimum, compensation of cell loss. A second potential mechanism refers to LRP1 as a member of the low-density lipoprotein (LDL)-receptor gene family that shares structural elements and multiple functions and might account for compensatory effects [51]. In addition to LRP1, LRP3, LRP4, and LRP6 are highly expressed in OPCs [9]. LRP2 has been suggested to mediate OPC proliferation and migration in connection with sonic hedgehog [52]. These properties identify the LRPs as promising candidates for the substitution of specific LRP1 functions after deletion in our new mouse model. To name one function, LDL-receptor family members, especially LRP1, serve as the main receptors for cholesterol uptake into oligodendrocytes with the help of apolipoproteins such as ApoE [53]. Cholesterol itself is critically involved in the maturation of oligodendrocytes and induces myelin-specific gene expression, transports myelin proteins, and participates in internode formation [54,55,56]. 

## 5. Conclusions

In summary, our novel triple transgenic mouse model provides new insights into the field of LRP1 and its functions in oligodendrocytes. We found that LRP1-deficiency induced postnatally in NG2-expressing OPCs led to progressive loss of recombined LRP1 KO oligodendrocytes over time. We conclude that LRP1-deficient OPCs are vulnerable and hardly survive. We propose a mechanism of cell death due to a phenotype susceptible to damage when LRP1 is deleted in the oligodendrocytic lineage. This was supported by observations in OPC cultures from cortical tissue, where only a few KO cells could be cultivated that hinted at a modified myelination behavior. In a further step, we found in an initial study that EAE can be elicited in the triple transgenic mouse line, where individual KO animals seemed to be clearly affected, yet without statistical significance. This presumably reflects the fact that most LRP1-deficient OPCs had been eliminated by the time the assay was carried out. Our model offers the perspective to test the roles of LRP1 by inducing recombination close to the commencement of inflammatory diseases. This experimental design might help to establish the role of LRP1 in myelin pathology in future studies.

## Figures and Tables

**Table 1:** Genotyping primers and amplified product sizes.

Gene	Primer Sequence	Product Size	Source
LRP1	for 5′-CATACCCTCTTCAAACCCCTTCCTG rev 5′-GCAAGCTCTCCTGCTCAGACCTGGA	WT: 291 bp KO: 350 bp	Jackson Laboratory
NG2-Cre (NGCE)	for 5′-GGCAAACCCAGAGCCCTGCC wt rev 5′-GCTGGAGCTGACAGCGGGTG Cre-ERT2 rev 5′-GCCCGGACCGACGATGAAGC	WT: 557 bp KO: 829 bP	[21]
Rosa26-GCaMP3	for wt 5′-CTCTGCTGCCTCCTGGCTTCT wt rev 5′-CGAGGCGGATCACAAGCAATA for KI 5′-CACGTGATGACAAACCTTGG rev KI 5′-GGCATTAAAGCAGCGTATCC	WT: 327 bp KO: 245 bp	[22]

## Supplementary Materials

The following are available online at https://www.mdpi.com/2073-4409/8/12/1550/s1, Figure S1: Myelinated artificial fibers were examined for the length and the number of myelin sheaths in LRP1-deficient and in control oligodendrocytes, Figure S2: Immunohistochemical stainings to detect LRP1 and GFP and to verify the staining specificity by secondary antibody controls, Figure S3: Exemplary analysis of cell numbers per area to characterize oligodendrocyte-specific lineage markers alternatively.
