# Supplementary material for: Conditional Deletion of LRP1 Leads to Progressive Loss of Recombined NG2-Expressing Oligodendrocyte Precursor Cells in a Novel Mouse Model
Source: Cells. 2019 Nov 30;8(12):1550. doi: 10.3390/cells8121550 (PMC6953036; doi:10.3390/cells8121550)
Supplement: Supplementary file 1 [file cells-08-01550-s001.pdf]

cells

Article

Conditional deletion of LRP1 leads to progressive loss of recombined NG2-expressing oligodendrocyte precursor cells in a novel mouse model

Supplementary Materials and Results

Methods

Immunopanning

The immunopanning procedure was performed as previously described by Ulc et al. [48]. Shortly, tamoxifen-treated KO and control animals were used at the age of P6-P9. The cortices were dissected and single cell suspensions were generated via tissue incubation in dissociation medium (MEM (Gibco by Thermo Fisher Scientific), 30U/ml Papain (Worthington, Columbia, NJ), 0.24mg/ml L-Cystein (Sigma), 40µg/ml DNase (Worthington)) for 1h at 37°C. The addition of an equal amount of ovomucoid (L15, 1mg/ml soybean trypsin inhibitor, 50µg/ml BSA fraction V (Sigma), 40µg/ml DNase I (Worthington)) and trituration stopped the dissociation and the cells were centrifuged at 100 x g for 10min. The supernatant was centrifuged for another 5min. Afterwards the remaining supernatant was discarded and sedimented cells of both steps were triturated in immunopanning buffer (PBS, 200µg/ml BSA, 5µg/ml Insulin (Sigma)). Then, the negative selection on a BSL-coated dish (BSL: Vector Laboratories, Biozol, Eching, Germany) was performed for 15min. After elimination of contaminating cell types, the remaining free-floating cells were positively selected for 45min via an antibody against PDGFRα (CD140a, rat, Biolegend, San Diego, CA), coated to a dish that was previously incubated with an anti-rat secondary antibody (Jackson ImmunoResearch Laboratories Inc.). With the help of this PDGFRα-antibody preferentially OPCs were isolated. Using a cell scraper (Sarstedt, Nümbrecht, Germany) cells were detached into oligodendrocyte medium (DMEM (Gibco), 2% B27, 1% SATO, 1% P/S, 1% L-Glutamine, 1% N-acetyl-cysteine, 5µg/ml Insulin, 1ng/ml D-Biotin, 2µg/ml Forskolin (Sigma); SATO: DMEM (Gibco), 10mg/ml BSA fraction V, 10mg/ml apo-transferrin, 6.25µg/ml progesteron, 1.61mg/ml putrescine, 4µg/ml sodium selenite (Sigma)). OPCs were then further cultivated for another 7-10 days in oligodendrocyte medium with PDGF-AA (10ng/ml) and NT3 (5ng/ml) (Peprotech, Rocky Hill, NJ) in order to expand the OPC population in PDL-coated (10µg/ml, Sigma) cell culture flasks (T75) at 7.5% CO_2. Every 2-3 days half medium exchange was done and additionally new growth factors were added daily.

Plating and Cultivation of OPCs on artificial fibers

Prior to plating and cultivating the OPCs on artificial fiber inserts composed of poly-L-lactic acid (Electrospinning Company, Didcot, UK) [47], the fibers were prepared and activated. After an initial incubation with 70% ethanol for 10-15min and three washes with sterile distilled water, 1h of PDL-coating (10µg/ml, Sigma) at 37°C followed. Finally the inserts were washed with myelination medium (DMEM/Neurobasal medium (1:1, Gibco), 2% B27, 1% P/S, 1% SATO, 1% ITS supplement, 5µg/ml N-acetyl-cystein, 10ng/ml D-Biotin (Sigma); SATO: DMEM (Gibco), 10mg/ml BSA fraction V, 6µg/ml progesteron, 1.61mg/ml putrescine, 40µg/ml T_3, 40µg/ml T_4 (Sigma)). Now the OPCs were detached from the cell culture flasks. First, one wash with PBS was performed prior to addition of

5ml Trypsin/EDTA (Gibco). After incubation for 4min at 37°C an equal amount of ovomucoid was added to stop the dissociation and detachment of the cells. The cell suspension was then transferred into a 15ml reaction tube and centrifuged at 1,000xg for 5min. Supernatant was discarded, myelination medium was added and the sedimented cells were triturated with different tips (1000µl, 200µl) and cannulas (23G, 21G, B. Braun) three times each for improved dissociation. A number of 50,000 cells (in a volume of 230µl myelination medium) was adjusted to fiber inserts (previously filled with 1.5ml myelination medium), placed in a 12-well-plate. The OPCs were allowed to differentiate and myelinate the coated fibers in the next 14 days at 7.5% CO_2 with complete medium exchange every 2-3 days.

Immunocytochemical staining of myelinating oligodendrocytes on artificial fibers

The immunocytochemical staining was performed as previously described [48]. Culture medium was discarded first, followed by two washes with PBS. Next the oligodendrocytes were fixed with 4% PFA (Carl Roth) for 15min. After another two to three washes with PBS the cells were incubated with PBS + 0.1% Triton X-100 (AppliChem, Darmstadt, Germany) to permeabilize the cells for 10min at room temperature. Then the incubation with primary antibodies against MBP (1:250, rat, BioRad) and GFP (1:300, rabbit, Millipore) diluted in PBS was performed at 4°C over night. The next day, three washes with PBS for 5min each were done before the incubation with the secondary antibody against rat and rabbit (Jackson ImmunoResearch Laboratories Inc.) followed. Therefor the antibodies were diluted in PBS, adjusted to the cells and stored for 1h at room temperature impervious to light. After three final washes with PBS the inserts were carefully removed from the wells and fibers were covered with Fluoromount G (Southern Biotech, Birmingham, AL) and 13mm coverslips (Thermo Fisher Scientific). After 24-48h of hardening of the covering medium the cells were imaged.

Imaging

Immunocytochemical stainings of myelinating oligodendrocytes in the fiber assay were imaged via an LSM 510 Meta (Zeiss). Z-stacks with a distance of 0.37µm were recorded until the complete myelin membrane of the object was scanned.

Quantification

Quantification of myelinated artificial fibers was done via ImageJ/FIJI. Completely myelinated sections were identified with the help of z-projections and the length of the internodes was measured via the segmented line tool and was documented in µm. Additionally to the mean length, the number of internodes per oligodendrocyte was analyzed.

Statistics

For the analysis of myelination of artificial fibers GraphPad Prism was used. Experiments were performed with N=2 and n=4.

Results

Artificial fiber assay and analysis of in vitro myelination of LRP1-deficient OPCs

To investigate myelination behavior of LRP1-deficient oligodendrocytes, we decided to perform an *in vitro* artificial fiber myelination assay. Therefore OPCs were isolated and cultivated via an immunopanning procedure. Afterwards the OPCs were plated on PDL-coated artificial fibers and were allowed to differentiate and to myelinate the fibers for 14 days *in vitro* (Figure S1 A+B). The

myelination behavior of the cells was examined with focus on numbers of internodes per cell and lengths of internodes in mean or per single cell.

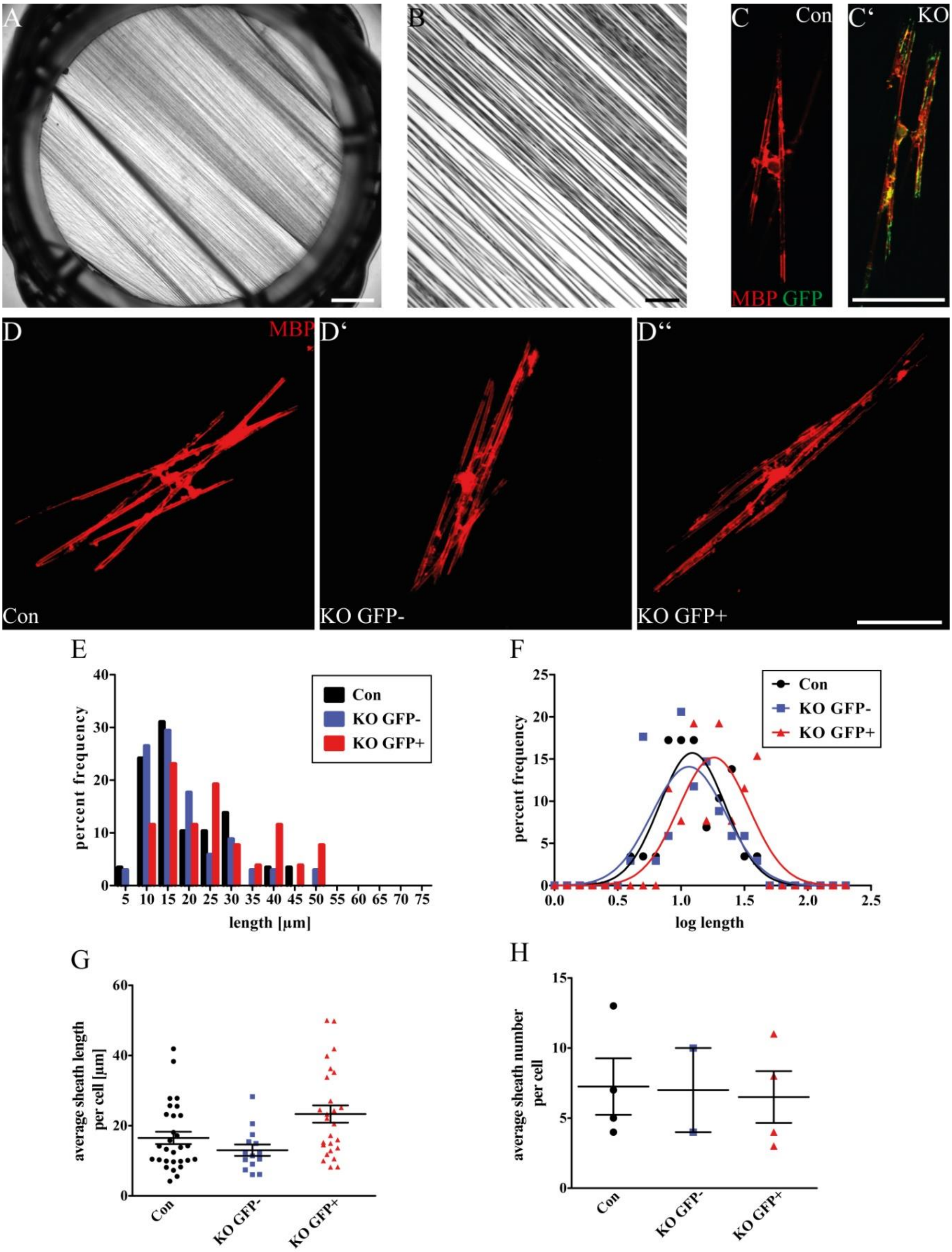

Figure S1: Myelinated artificial fibers were examined for the length and the number of myelin sheaths in LRP1-deficient and in control oligodendrocytes

A Insert with artificial fibers (scale bar: 2000µm). **B** Higher magnification of artificial fibers (scale bar: 100µm). **C** Immunocytochemical staining against MBP demonstrating myelinated areas of artificial fibers within control (con, GFP-negative) and KO condition (GFP-positive). (Scale bar: 50µm). **D**

Representative pictures of myelinating cells from control and KO tissue: two controls (Con, KO GFP-) and one knockout (KO GFP+) (scale bar: 100µm). **E** The histogram with myelin sheath lengths in µm reflects the lengths of myelin sheaths. **F** Gaussian curve fit in a log length graph. **G** Average sheath length per single cell. **H** The average sheath number per single cell. N=2, n=4.

To address this point, the myelinating cells were immunocytochemically stained with antibodies against MBP and GFP (KO cells: GFP-positive cells, control cells: GFP-negative cells) (Figure S1 C+D). In agreement with the interpretation of an increased fragility of LRP1-deficient OPCs only a minor population of recombined cells withstood the cultivation procedure. Consequently, less than 1% of the myelinating oligodendrocytes expressed GFP, which we did not consider sufficient for a rigorous statistical analysis. With this reserve in mind, we saw elongated myelin sheaths or rather internodes in the length plot, the log length plot and the mean internode length of the investigated recombined cells within the KO condition compared to the control (Figure S1 E-G). However, the number of internodes was not impaired under these conditions (Figure S1 H). These findings suggested a modified myelination in LRP1-deficient cells, consistent with the assumption that LRP1 might be involved in oligodendrocytic functions *in vitro*.

Comparison of control and recombined tissue by immunohistochemistry with distinct markers

Recombined tissue displayed clearly a larger number of recombined GFP-expressing cells than the control (Figure S2, GFP-labelling). The control experiment shows that secondary antibodies alone did not yield significant signals in the tissue sections (Figure S2, secondary antibody labelling).

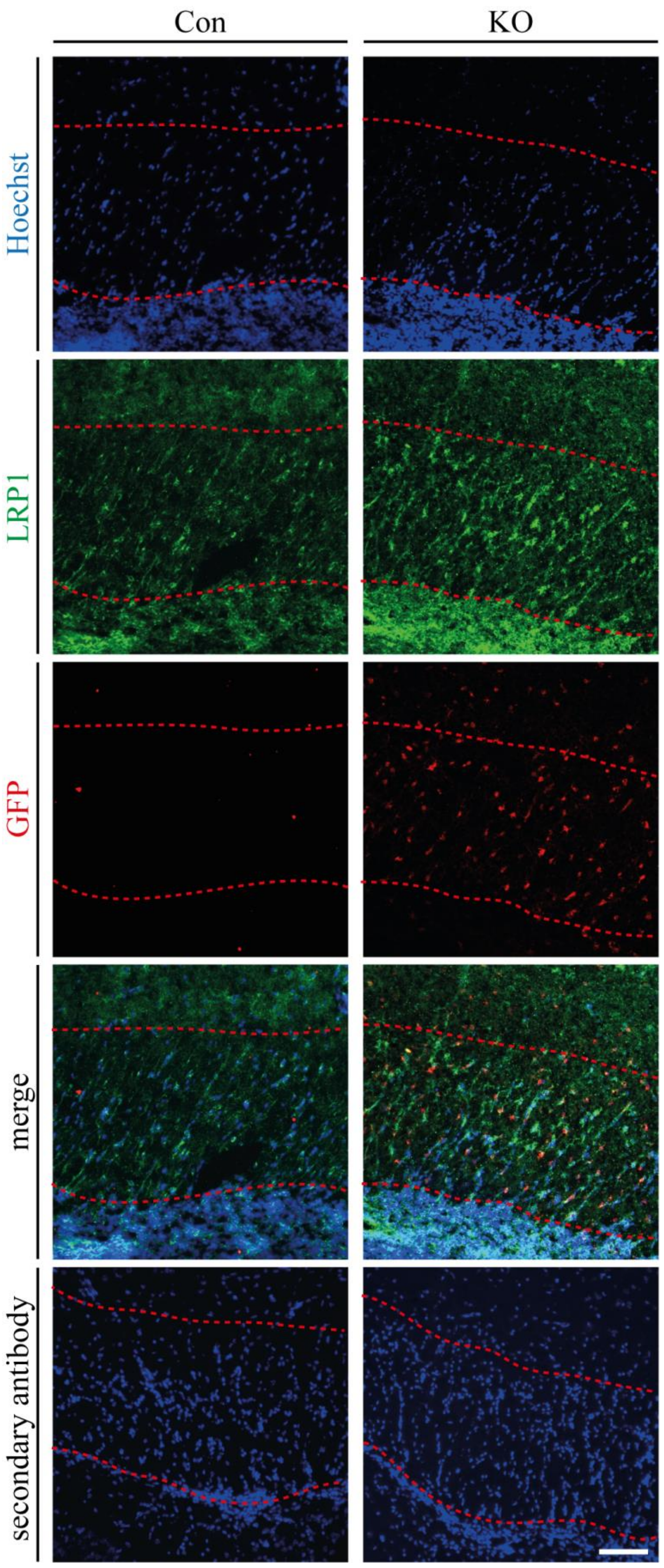

Figure S2: Immunohistochemical stainings to detect LRP1 and GFP and to verify the staining specificity by secondary antibody controls.

Representative staining in P7 tissue with focus on Corpus callosum (within red lines). Cell nuclei were detected with Hoechst dye. LRP1 was labeled with Cy2-coupled donkey anti-rabbit antibody and GFP was identified by Cy3-coupled donkey anti-goat antibody. Channels are presented as single and merge images. Please note that cells that do not pertain to the NG2-lineage are not recombined and hence conserve LRP1-expression. Additionally the specificity of the staining was

evaluated by immunohistochemical stainings with secondary antibodies only (merge images shown). Scale bar: 100µm.

Cellular characterization of oligodendrocyte lineage-associated cells

We have scored the numbers of Olig2- or PDGFRα-expressing cells with reference to the total number of cell nuclei detectable in the section, as revealed by staining with the Hoechst dye (Figure 6). As an alternative approach, we have probed the number of labeled cells with reference to the surface area in mm². Differences that emerged when using the number of nuclei was used as basis (Figure 6) did not substantiate when the latter parameter was applied. However, this approach does not consider variations in cell density.

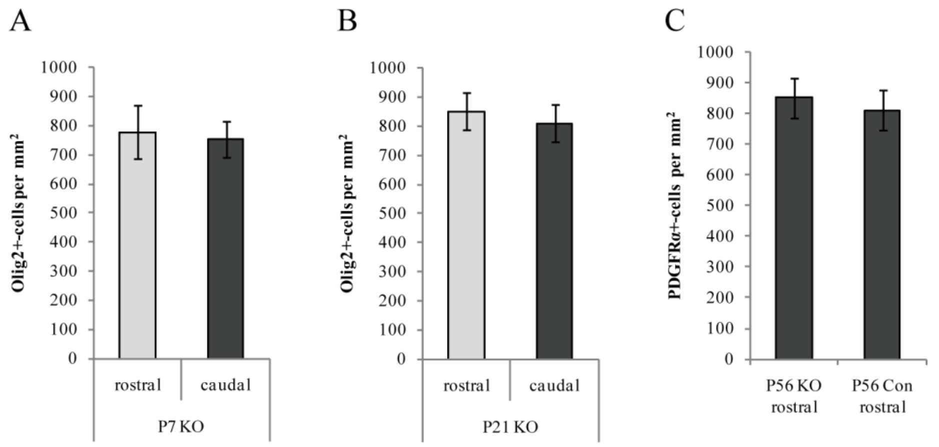

Figure S3: Exemplary analysis of cell numbers per area to characterize oligodendrocyte-specific lineage markers alternatively.

A Number of Olig2-positive cells normalized to the analyzed area in mm² in the KO at P7, differening between the rostral and caudal Corpus callosum. **B** Number of Olig2-positive cells normalized to the analyzed area in mm² in the KO at P21, separating between the rostral and the caudal part of the Corpus callosum. **C** Number of PDGFRα-positive cells normalized to the analyzed area in mm² at P56. Rostral parts of the Corpus callosum were compared. Data are expressed as mean ± SEM. N=3-4, n=9-12 per rostral and caudal part. At least 200-1200 cells per area were counted. Depending on normally or not normally distributed data, Student's *t*-test or Mann-Whitney U-test were used for the evaluation within the individual ages.
